# Membrane-Type 4 Matrix Metalloproteinase (MT4-MMP) Modulates Water Homeostasis in Mice

**DOI:** 10.1371/journal.pone.0017099

**Published:** 2011-02-11

**Authors:** Manakan B. Srichai, Heloisa Colleta, Leslie Gewin, Linsey Matrisian, Ty W. Abel, Naohiko Koshikawa, Motoharu Seiki, Ambra Pozzi, Raymond C. Harris, Roy Zent

**Affiliations:** 1 Department of Medicine, Veterans Administration Hospital, Nashville, Tennessee, United States of America; 2 Division of Nephrology, Department of Medicine, Vanderbilt University Medical Center, Nashville, Tennessee, United States of America; 3 Department of Pathology, Vanderbilt University Medical Center, Nashville, Tennessee, United States of America; 4 Department of Cancer Biology, Vanderbilt University Medical Center, Nashville, Tennessee, United States of America; 5 Department of Cell and Developmental Biology, Vanderbilt University Medical Center, Nashville, Tennessee, United States of America; 6 Division of Cancer Cell Research, Institute of Medical Science, University of Tokyo, Minato ku, Tokyo, Japan; University of South Florida College of Medicine, United States of America

## Abstract

MT4-MMP is a membrane-type metalloproteinase (MMP) anchored to the membrane by a glycosyl-phosphatidylinositol (GPI) motif. GPI-type MT-MMPs (MT4- and MT6-MMP) are related to other MT-MMPs, but their physiological substrates and functions *in vivo* have yet to be identified. In this manuscript we show that MT4-MMP is expressed early in kidney development, as well as in the adult kidney, where the highest levels of expression are found in the papilla. MT4-MMP null mice had minimal renal developmental abnormalities, with a minor branching morphogenesis defect in early embryonic kidney development and slightly dysmorphic collecting ducts in adult mice. Interestingly, MT4-MMP null mice had higher baseline urine osmolarities relative to wild type controls, but these animals were able to concentrate and dilute their urines normally. However, MT4-MMP-null mice had decreased daily water intake and daily urine output, consistent with primary hypodipsia. MT4-MMP was shown to be expressed in areas of the hypothalamus considered important for regulating thirst. Thus, our results show that although MT4-MMP is expressed in the kidney, this metalloproteinase does not play a major role in renal development or function; however it does appear to modify the neural stimuli that modulate thirst.

## Introduction

The matrix metalloproteinases (MMP) are a 23 member family of zinc-binding endopeptidases that primarily function as promoters of extracellular matrix degradation and are collectively responsible for tissue remodeling during embryogenesis, organogenesis, tissue regeneration, wound healing and many other physiologic and pathologic conditions. All MMPs are multidomain enzymes with a common conserved domain structure consisting of a prodomain, a catalytic domain, a hinge region, and a hemopexin-like domain. Seventeen members are secreted as soluble enzymes while 6 members are membrane-type MMPs (MT-MMP), which are either associated with the cell membrane by a type I transmembrane domain (MT1-MT3 and MT5) or are glycosyl-phosphatidyl inositol (GPI)-anchored (MT4 and MT6). While MT1-, MT2-, MT3- and MT5-MMP display potent proteolytic activity towards ECM components, [1], [2], the GPI-anchored MT-MMPs, including MT4-MMP and MT6-MMP do not possess clear proteolytic activity towards ECM substrates.

MT4-MMP has the least degree of sequence identity to the other MMP family members and little is known about its function [3], [4]. It lacks a cytoplasmic tail suggesting it may have different functional capabilities, and consistent with this it is unable to activate pro-MMP2 [5]. The only well-defined substrate for MT4-MMP is ADAMTS4, which has tumor necrosis factor-alpha convertase activity; however the functional significance of this interaction is unknown. Although studies have shown a role for MT4-MMP in promoting cancer metastasis in breast [6] and glial cancers [7], the mechanisms for these actions are still undetermined.

MT4-MMP is expressed in many areas of the body with highest levels seen in the brain (particularly in the cerebrum), colon, ovary, and testis [3], [8]. However, we have determined that there is also high expression of MT4-MMP in the kidney papilla (current manuscript). Numerous MMPs are expressed in the kidney in a poorly characterized and complex pattern. The only MMP shown to play a role in renal development *in vivo* is MT1-MMP, where the null mice develop dysmorphic and dysgenic kidneys [9]. Although MMPs do not play a major role in development, they have been implicated in both acute kidney injury as well as in chronic kidney diseases, including polycystic kidney disease, glomerulosclerosis/tubulointerstitial fibrosis, chronic allograft nephropathy, diabetic nephropathy, and renal cell carcinoma [10]. Most of these studies demonstrated alterations in expression of various MMPs, however there are a few in vivo studies indicating beneficial effects of blocking MMP activity in different models of kidney injury [11], [12], [13]. Based on the relative scarcity of data on the role of MT4-MMP, particularly within the kidney, we characterized MT4-MMP expression within the kidney and describe interesting abnormalities associated with water homeostasis observed in MT4-MMP deficient mice.

## Methods

### MT4-MMP null mice and general reagents

All procedures on animals were approved by the Institutional Animal Care and Use Committee of Vanderbilt University and conducted according to the NIH Guide for the Care and Use of Laboratory Animals. This was covered by protocol number M/04/219 from Vanderbilt Medical Center.

MT4-MMP null mice were described previously [8] and because part of the mouse MT4-MMP genomic locus was substituted with the bacterial LacZ gene we were able to monitor of MT4-MMP expression by tracking β-gal expression. MT4-MMP null mice were backcrossed onto the C57/BL6 strain (10^th^ generation) for all experiments. C57/BL6 mice were purchased from Harlan Laboratories. Genotyping of MT4-MMP mice was performed by standard PCR with the following 3 primers: 1) MT4 forward 5′-CCAGGTGGCAGAGGTAAGTA-3′; 2) MT4 new forward 5′-AAGGTGCTGAAGGTTCGAGA-3′; and 3) MT4 reverse 5′- CAAGGAATAAGGTGAGCTTCAGAA-3′, which yields a 180 bp wild-type band and a 350 bp knockout band. For urine concentration studies, DDAVP (Sigma, St. Louis, MO) was administered intraperitoneally as a onetime 10 ng dose and urine was collected following 3 hours of water deprivation post-DDAVP.

### Histology and Immunohistochemistry

All kidneys were fixed in 4% paraformaldehyde, paraffin-embedded and 5 µm sections were cut for Hematoxylin & Eosin or Periodic acid Schiff staining. Antibodies used included rabbit anti-MT4 (1∶200, Biovision, Mountain View, CA), rabbit anti-AQP2 (1∶250, Alpha Diagnostics, San Antonio, TX), rabbit anti-AQP1 (1∶250, Chemicon International, Billerica, MA), and rabbit anti-ENaCβ (gift from Mark Knepper, NIH).

### β-gal staining

Adult mice were perfused-fixed in 4% paraformaldehyde, then fixed for an additional 2 hours in 4% paraformaldehyde. Brain and kidney sections were sliced and incubated in X-gal buffer (5 mM potassium ferrocyanide, 5 mM potassium ferricyanide, 2 mM MgCl_2_, 0.01% deoxycholate acid, 0.02% NP-40, and 0.1% X-gal) at 30°C overnight. Following PBS washing, tissues were paraffin-embedded and sectioned. Brain sections were counterstained with hematoxylin and eosin to better define brain structure. Kidney sections were left unstained.

### Organ Culture

Kidneys from 12 day MT4-MMP-null or C57 embryos were removed and grown on culture dishes in DMEM/F12 10% FBS for 48 hr. Following attachment and growth, kidneys were stained for E-cadherin (BD Biosciences, San Jose, CA) followed by FITC-anti-rabbit and visualized using a fluorescent microscope (Olympus, Center Valley, PA).

### MT4-MMP null cell derivation

Collecting duct cells were isolated from MT4-MMP null and wildtype mice following the methodology described by Husted et al. [14]. The MT4-MMP null cells were transduced with a human c-DNA of MT4-MMP and sorted by flow cytometry for MT4-MMP expression.

### Immunoblotting

The collecting duct cells were lysed with RIPA buffer after which the lysates were clarified by centrifugation and 20 µg total protein was electrophoresed onto an 10% SDS-PAGE and subsequently transferred to nitrocellulose membranes. Membranes were blocked in 5% milk/TBS Tween and then incubated with the MT4-MMP primary antibody followed by an HRP-conjugated secondary antibody. Immunoreactive bands were identified using enhanced chemiluminescence according to the manufacturer's instructions.

### Quantification of glomerular number

Glomeruli were counted as previously described [15], [16]. Briefly, individual kidneys were isolated from adult mice (>8 weeks of age) and minced into 2-mm cubes. Fragments were incubated in 5 ml of 6 M HCl at 37°C for 90 min. Following incubation, tissues were homogenized through repeated pipetting and 25 ml of H_2_O added followed by overnight incubation at 4°C. Tissues were stored at 4°C. To count glomeruli, 5×1 ml of this solution was counted in a 35-mm counting dish.

### Metabolic studies

Mice undergoing metabolic studies were acclimated to metabolic cages (Hatteras, Cary, NC) for at least two days until weights stabilized. Twenty-four hour water, food intake and urine output were recorded. For chronic water loading, mice were fed high water gel food [17] consisting of 19 ml of gel diet containing 14.8 ml of water, 4 grams powdered food (AIN-93G, Test diet, Richmond, IN) and 3.8% porcine gelatin (Sigma-Aldrich, St. Louis, MO), daily for 1 week. Following chronic water loading, mice were subjected to acute water loading and administered 2 ml water intraperitoneally. Animals were then water-restricted and urines collected hourly for urine osmolarity. Urine osmolarity was measured with a urine osmometer (Precision Systems, Natick, MA). i-STAT EC-8+ cartridges (Heska, Loveland, CO) were used to measure whole blood BUN and sodium (Na).

### Statistical analysis

Data were analyzed using GraphPad Prism 5 for Windows software. For statistical analysis of all parameters, the Mann-Whitney t tests were used. For branching count analysis, the number of branches from each embryo (average 6 per group) were counted and compared between groups. Mann-Whitney test statistical analysis was performed. For total glomerular number, the average number per kidney was extrapolated mathematically from the mean of five counts. At least 3 kidneys from each genotype were counted and statistical analysis performed using the Mann-Whitney test.

## Results

### MT4-MMP is expressed in the kidney

**Figure 1:**
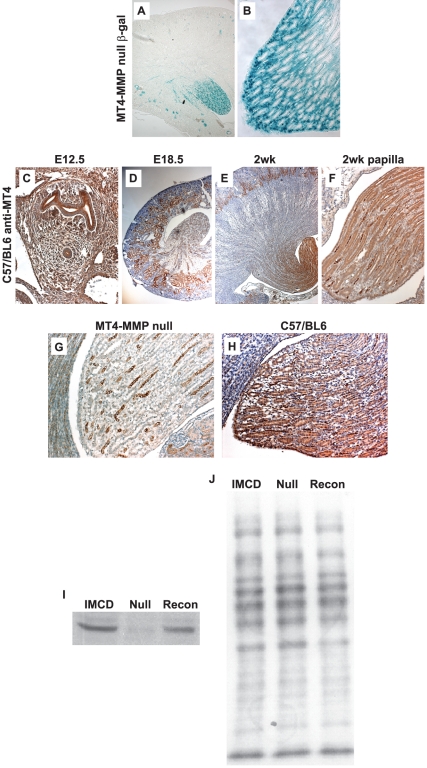
MT4-MMP localization in the mouse kidney. (**A–B**) β-galactosidase staining kidneys from 5week old MT4-MMP null mice shows staining in the cortex and medulla (A, 50X DIC) with the most intense staining seen in the papilla (B, 200X DIC). (**C–F**) Staining with a specific antibody directed against MT4-MMP in wildtype mice shows expression throughout the metanephric mesenchyme and ureteric bud at E12.5 (C, 100X). MT4-MMP expression is abundant in the cortical regions during embryonic development (E18.5, D, 50X), but by 2weeks of age, most of the expression is localized to the papilla regions (E–F, 50X and 200X, respectively). (**G–H**) Staining with a specific antibody directed against MT4-MMP in MT4-MMP null and wildtype mice, demonstrating specificity of the antibody for MT4-MMP (200X). (**I–J**) Immunoblot of inner medullary collecting duct cells derived from wild type mice (IMCD), MT4-MMP null mice (Null) or IMCD cells from MT4-MMP null mice reconstituted with MT4-MMP (Recon), illustrating specificity of the antibody for MT4-MMP (I). Ponceau staining was performed on the nitrocellulose of the immunoblot to demonstrate equal loading of the lysates (J).

High levels of MT4-MMP expression in the kidney were not observed in initial studies where MT4-MMP expression was studied in mice [3], [8]. However, characterization of adult MT4-MMP null mice with β-galactosidase knocked into the MT4-MMP locus showed that high levels of β-galactosidase were expressed within the papilla of the kidney (Figure 1A–B) with less expression in the cortex. To confirm these results, immunohistochemistry using an antibody specific for MT4-MMP was performed in the kidney from early development (E12.5) to adulthood. MT4-MMP is highly expressed within both the ureteric bud and metanephric mesenchyme at embryonic day 12.5 (Figure 1C). At E18.5 expression is seen in both the cortical and medullary tubules (Figures 1D) and by 2 weeks of age expression is still present within the kidney cortex; however, the majority of expression is localized to the collecting ducts in the papilla (Figures 1E–F). The specificity of the MT4-MMP antibody was confirmed by performing immunohistochemistry on wild type and MT4-MMP null kidneys. As shown in figure 1G there is only background none specific staining in the MT4-MMP null kidneys, while there is intense staining in the wild type kidneys (Figure 1H). To further confirm the specificity of the antibody we performed immunoblots on inner medullary collecting duct cells derived from wild type as well as MT4-MMP null mice. As seen in figure 1I the antibody was immunoreactive with lysates from cells derived from wild type mice and cells from MT4-MMP null mice that were transduced with human MT4-MMP. No immunoreactivity was observed in the lane loaded with lysates of MT4-MMP null cells. Equal loading of protein in each lane was verified by Ponceau staining of the membrane (Figure 1J). Thus MT4-MMP expression in the kidney changes during development, but the predominant region of expression both in early development and postnatally is the collecting system, which is derived from the ureteric bud.

**Figure 2:**
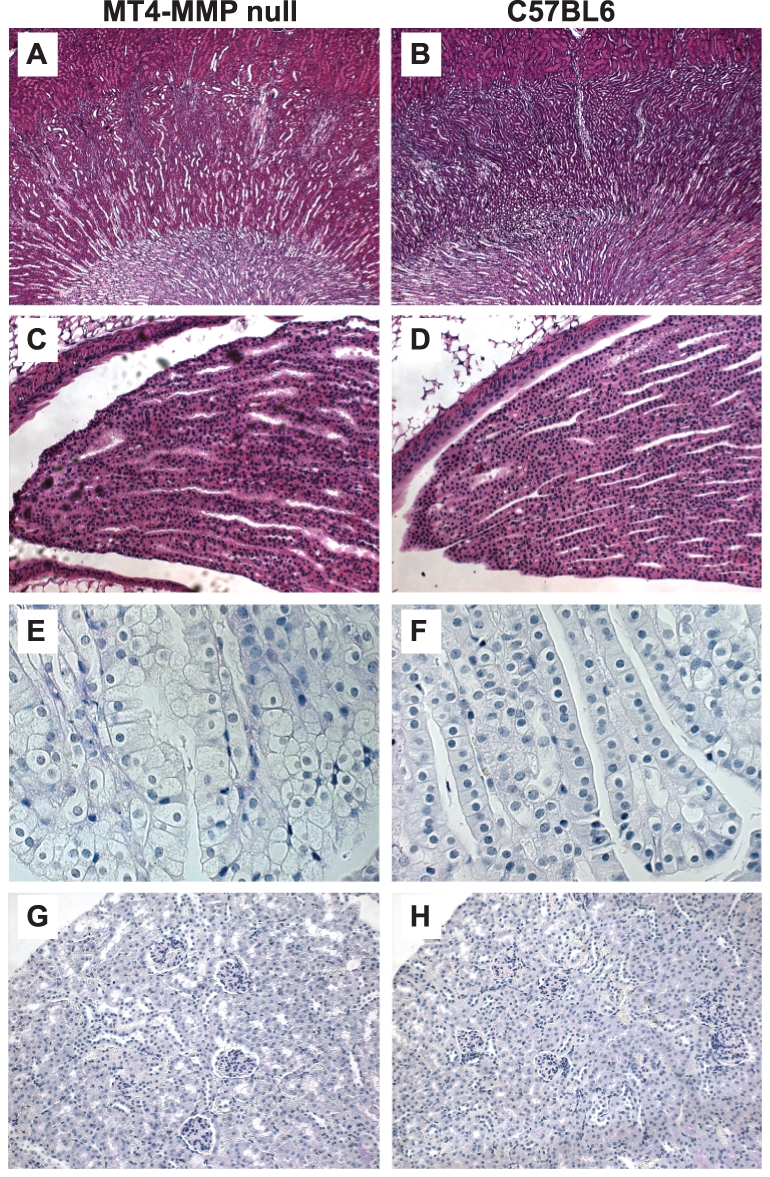
Adult MT4-MMP null have subtle abnormalities in the renal papilla. (**A–B**) No gross abnormalities are seen in low power (50X) images of H and E stained kidneys from MT4-MMP null mice (A) when compared to C57/BL6 controls (B). (**C–F**) Tubules in the deep papilla of MT4-MMP null kidneys are less tightly packed and show subtle evidence of disorganization (C, E). C–D, H&E, 200X; E–F, PAS, 400X. (**G–H**) The cortex of MT4-MMP null kidneys appear morphologically normal with no apparent decrease number of glomeruli, PAS, 200X.

### MT4-MMP null adult kidneys have subtle abnormalities in the deep papilla of the kidney

To determine whether deleting MT4-MMP in mice resulted in a renal phenotype, we performed histological analysis on adult kidneys. There were no gross anatomical abnormalities in the kidney cortex or medulla in the MT4-MMP null mice (Figure 2A–B); however, in the papilla there were subtle defects of collecting duct cellular organization (Figure 2C–F) and a decrease in density of tubules within the papilla. The cortex appeared similar in wildtype and MT4-MMP null mice (Figure 2G–H). No differences in Ki67, a marker of proliferation, were seen (data not shown).

**Figure 3:**
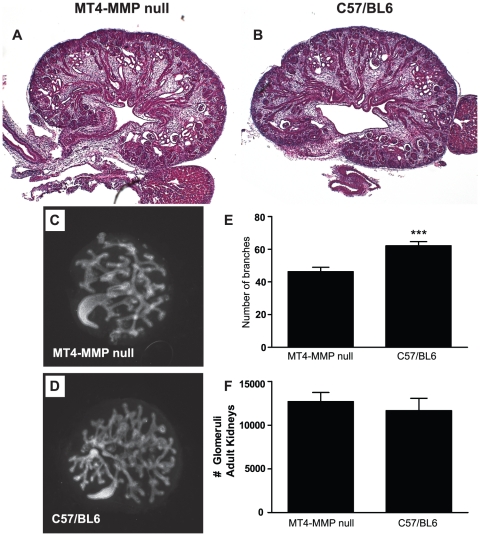
MT4-MMP null mice have a mild early embryonic branching morphogenesis defect when cultured *in vitro*. (**A–B**) MT4-MMP null kidneys at P0 do not show a branching defect when compared to C57/BL6 control kidneys 50X, H&E. (**C–D**) E12.5 kidneys were dissected and grown in organ culture for 72 hours and stained for E-cadherin. Fewer branches are seen in MT4-MMP null kidneys (C) compared to C57/BL6 controls (D). (**E**) Quantification of branching number in kidney organ culture reveals MT4-MMP null kidneys have significantly less branching, ** p<0.05. (**F**) Quantification of glomerular number in adult kidneys reveals no differences between the two genotypes.

Kidneys were assessed at different developmental stages from E12.5 to P1. There were no significant developmental abnormalities present in the MT4-MMP null kidneys during embryogenesis (data not shown), and this was confirmed at P0 (Figure 3 A–B). When embryonic day 12.5 kidneys were cultured *in vitro* for 72 hours, the MT4-MMP null kidneys displayed a mild but statistically significant decrease in the number of branches as compared to controls (Figure 3C–E). To define whether this subtle *in vitro* branching phenotype resulted in a difference in nephron number in adult kidneys, glomerular counts were performed on 8week old kidneys. No difference was evident between MT4-MMP null and control kidneys (Figure 3F). Thus, although a mild branching defect is present in an *in vitro* kidney culture model early in development only a subtle disorganization in the renal papilla is seen *in vivo* in the adult kidney. Thus MT4-MMP appears to play a minor role in normal renal development.

**Figure 4:**
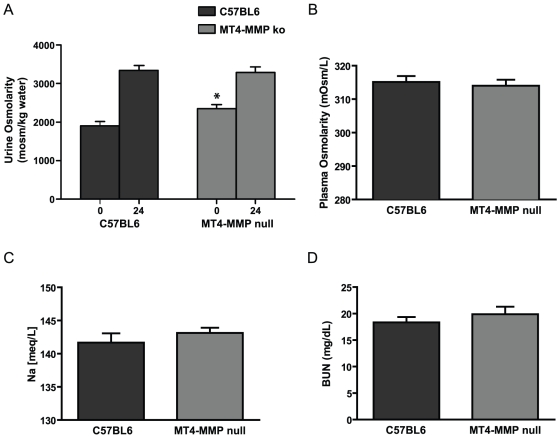
MT4-MMP null mice have increased urine osmolarity compared to control mice. (**A**) Urine osmolarity obtained at baseline (0) and following 24 hr water deprivation (24) reveals that MT4-MMP null mice have higher baseline urine osmolarity (*p = 0.0164) vs C57/BL6 time point 0. Both groups of animals appropriately concentrate their urines following water deprivation. (**B**) Plasma osmolarity, (**C**) Plasma sodium and (**D**) Blood urea nitrogen were the same in both groups.

*MT4-MMP null mice have increased urine baseline urine osmolarities.* Although the phenotypic changes in the MT4-MMP deficient kidneys were subtle, we observed that the urine of the MT4-MMP null mice was consistently more concentrated than wildtype mice. The average baseline osmolarity of wildtype mice was 1800 mosm/kg, but it was significantly higher (2400 mosm/kg) in the MT4-MMP null mice (Figure 4A). However, following 24 hr water deprivation both groups of animals exhibited maximal urinary concentration, with an osmolarity of approximately 3400 mosm/kg [18]. Similar results were noted when mice were administered desmopressin; a synthetic replacement for vasopressin (data not shown). To determine whether these differences in urine osmolarity were due to alterations in serum osmolarity or sodium these parameters were measured. Both of these parameters (Figure 4B and C) were similar between the two groups. Serum blood urea nitrogen (BUN) levels (Figure 4D) were also similar in the wild type and MT4-MMP null mice demonstrating that the renal function of these mice was normal.

### Increased urinary osmolarity in MT4-MMP mice is due to hypodipsia

**Figure 5:**
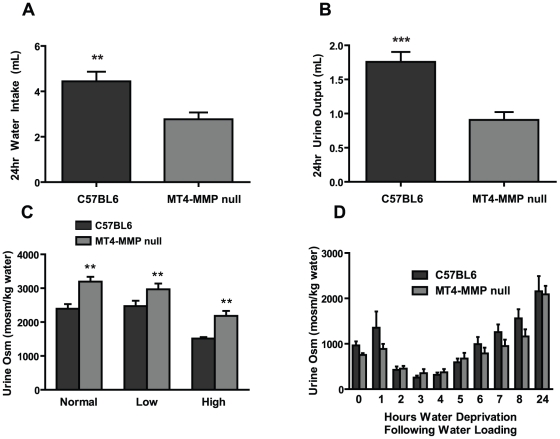
Metabolic studies show that MT4-MMP null mice dilute and concentrate urine normally. (**A**) 24 hour water intake (**p<0.01) and (**B**) 24 hour urine output is decreased in MT4-MMP null mice (**p<0.01). (**C**) Baseline urine osmolarity is always higher in MT4-MMP null mice compared to control (**p<0.05), regardless of dietary sodium content. (**D**) When mice were chronically water loaded for a week, MT4-MMP null mice appropriately diluted their urines (0). When 2 ml of water was injected intraperitoneally MT4-MMP null mice diluted and concentrated their urines in a similar fashion to C57/BL6 controls.

To define the mechanisms whereby the MT4-MMP null mice had consistently higher baseline urinary osmolarity, we placed them in metabolic cages to monitor both intake and output of water and solutes. The MT4-MMP null mice drank approximately half the volume of water of wild type controls and produced 50% less urine (Figures 5A and B). We next defined whether the MT4-MMP null mice exhibited abnormalities in salt or water handling by the kidney. Mice were fed diets containing normal salt (0.4% Na), low-salt (0.03% Na) or high-salt (3.15% Na) contents, and urine osmolarity was measured. Urine osmolarities were consistently higher in MT4-MMP null mice (Figure 5C) regardless of dietary salt intake, suggesting that these mice have no abnormalities in salt handling by their kidneys.

To assess whether MT4-MMP null mice could appropriately dilute their urine, mice were subjected to chronic water loading by administration of a high water gelled diet for one week after which they were administered an acute 2 ml intraperitoneal water load. The mice were then water restricted, and serial urine osmolarity was measured. Following chronic water loading, both groups of mice were able to establish similar baseline urine osmolarities of approximately 1000 mOsm/kg (Figure 5D, time 0). Within 3 hours of receiving an acute water load both groups of mice diluted their urines to approximately 300 mOsm/kg (Figure 5D). Subsequently, both groups appropriately concentrated their urines at similar rates over the following 15 hours. Thus the mice have no renal abnormalities with respect to diluting their urines after water loading.

**Figure 6:**
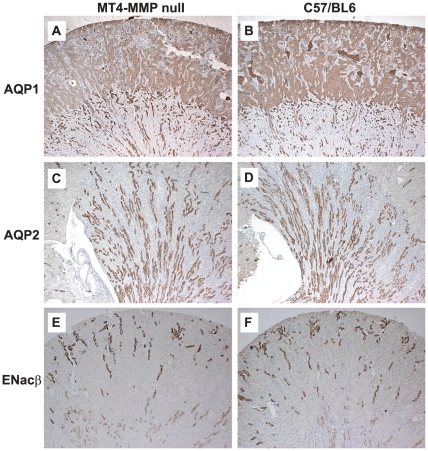
MT4-MMP null mice do not have abnormalities in aquaporin 1, 2 or ENaC expression. Immunostaining was performed for (**A–B**) Aquaporin I, (**C–D**) Aquaporin 2, and (**E–F**) ENaCβ (50X).

To define whether there were any alterations in expression of the principal water channels of the kidney or key regulators of sodium reabsorption in the collecting duct of the kidney, we performed immunostaining for aquaporin-1 (AQP1, Figure 6A–B), aquaporin-2 (AQP2, Figure 6C–D) and the epithelial sodium channel ENaC-β (Figure 6E–F). No obvious differences in expression of these proteins were defined between MT4-MMP null and control kidneys. These data further suggest that the MT4-MMP null kidneys function normally with respect to regulating sodium and water homeostasis.

### MT4-MMP is expressed in osmoreceptors located in the anterior hypothalamus of the brain

**Figure 7:**
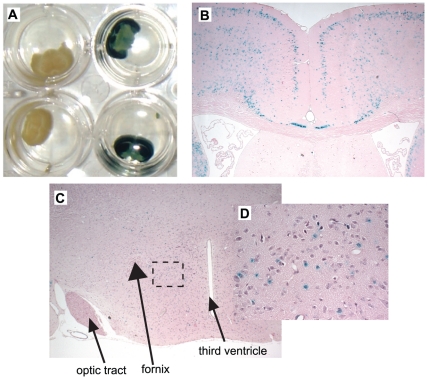
MT4-MMP is expressed in regions of the hypothalamus that regulate thirst. (**A**) β-gal staining of MT4-MMP null mouse brain reveals intense blue staining. No staining is present in the wildtype controls. (**B**) β-gal staining is mainly within the cortex of the MT4-MMP null mice (12.5X). (**C**) β-gal staining is present in scattered neurons within the hypothalamus (100X), including the anterior hypothalamic area and lateral hypothalamic area, which are known to regulate thirst. (D) Inset shows high power view (400X) of the hypothalamus revealing blue staining within neurons.

The data obtained from our metabolic cage studies suggested that the increased serum osmolality of the MT4-MMP mice was due to primary hypodypsia. We therefore performed β galactosidase staining to determine MT4-MMP expression in the anterior hypothalamus, which contains the thirst center of mice. As noted previously, β-galactosidase was highly expressed in the brain, especially the cortex (Figure 7A) where it was found in neurons (Figure 7B). Although the level of expression was less than in the cortex, scattered expression was found throughout the remainder of the cerebrum including the hypothalamus (Figure 7C–D). At high magnification, blue-staining was noted within neurons in the anterior hypothalamic area, which regulates drinking behavior. Thus it is plausible that MT4-MMP plays a role in the neural regulation of thirst.

## Discussion

MT4-MMP (MMP17) was discovered over 10 years ago [3], however little is known regarding its natural substrates, expression pattern and *in vivo* and *in vitro* functions. In this report, we show that MT4-MMP-null mice have higher baseline urine osmolarities and consume less water than wildtype controls. We demonstrate that although MT4-MMP is expressed in the developing and adult kidney, kidney development and function with respect to salt and water homeostasis in MT4-MMP-null mice is normal. Finally we show that MT4-MMP is expressed in areas of the anterior hypothalamus of the brain responsible for regulating thirst. These results suggest that although MT4-MMP does not play a major role in kidney development or function, it may modulate the sensation of thirst, which is regulated in the anterior hypothalamus of the brain.

Like many of the MMPs, MT4-MMP is expressed in the kidney but plays little role in renal development. Although our initial phenotyping of E12.5 MT4-MMP null embryonic kidneys grown on transwells showed a moderate branching phenotype, only subtle morphological abnormalities in the papilla of the kidney were noted. These results were similar to those seen for the gelatinases where mice deficient for MMP-2 or MMP-9 or both MMP-2 and MMP-9 do not have a renal phenotype, although *in vitro* organ cultures did demonstrate that MMP-9 might play a role in ureteric bud branching [19], [20], [21], [22]. MT1-MMP is the only MMP known to modulate renal development, and MT1-MMP null mice kidneys exhibit a moderate decrease in ureteric bud branching morphogenesis and a severe proliferation defect [9]. While it has been proposed that the lack of a renal phenotype in the various MMP-null mice could be due to functional redundancies, this is unlikely to be the case for MT4-MMP as it is structurally distinct from other MMPs, including the MT-MMPs. The catalytic domain of MT4-MMP only possesses 37% identity (50% similarity) with that of MT1-MMP and the catalytic domains of both GPI-linked MT-MMPs, MT4-MMP and MT6-MMP, are only 56% identical and 77% homologous, further suggesting substrate specificity for this MMP [23].

The MT4-MMP-null mice demonstrated an increase in urine osmolarity. This contrasts with the low urine osmolarities found in most urinary concentrating defects seen in mice where water channels or urea transporters are deleted. MT4-MMP null mice exhibited consistently higher urine osmolarities compared to wild-type control mice regardless of dietary salt content. However, MT4-MMP null mice subjected to overnight water restriction or to desmopressin in conjunction with 3 hours water deprivation were able to concentrate their urines to the expected maximal levels. In addition, following chronic water administration, MT4-MMP null mice were able to dilute their urines and excrete an acute water load at the same rate as controls, suggesting that their ability to maximally dilute their urines was intact. These findings indicated that despite higher baseline urine osmolarities, a renal concentrating defect was not present. These data strongly suggest that the mice had hypodipsia due to a primary disorder affecting the sensation of thirst [24].

The hypodipsia noted in the MT4-MMP null mice was not associated with abnormalities in serum electrolytes or an increase in plasma osmolarity. Furthermore, the MT4-MMP null mice did not have abnormalities in vasopressin secretion, as they could appropriately concentrate their urines in response to water deprivation. We also noted MT4-MMP expression in the anterior hypothalamic areas of the brain, which is known to mediate thirst. Thus the phenotype of the MT4-MMP-null mice is likely related to a yet undetermined role for MT4-MMP in regulating thirst, which is different from the mechanisms found in hypodipsic hypernatremia seen in conditions such as intrahypothalamic hemorrhage, primary neoplasms associated with the brain (craniopharyngioma, pinealoma, meningioma), hydrocephalus, and head injuries, which are associated with abnormal vasopressin secretion [25].

There are genetic models of mice that have abnormalities with drinking. For example mice that are null for the TRPV4 channel have hypodipsia and associated hyperosmolarity. This channel is expressed in the organum vasculosum of the lamina terminalis and subfornical organ, which sense osmotic pressure and project to neurons in the supraoptic and paraventricular nucleus of the hypothalamus. Urine osmolarity was not measured in *trpv4^-/-^* mice, but they were noted to have lower antidiuretic hormone levels compared to control mice [26], suggesting that TRPV4 is necessary for the normal response to changes in osmotic pressure and functions as an osmotic sensor in the CNS. This phenotype is different from MT4-MMP null mice, which do not have abnormalities with serum osmolality and MT4-MMP expression is not seen in the paraventricular nucleus (data not shown). Thus the underlying mechanisms for the hypodipsia between the TRPV4-null and MT4-MMP null mice are likely to be different.

In conclusion, we demonstrated that MT4-MMP is highly expressed in the mouse kidney and brain; however it only plays a minor role in renal development and function. Despite the normal kidney function, the MT4-MMP null mice demonstrate hypodipsia and consequently develop increased urine osmolarity, which we believe is likely due to disrupted thirst regulation within the anterior hypothalamus of the brain.
